# A risk score model of contrast-induced acute kidney injury in patients with emergency percutaneous coronary interventions

**DOI:** 10.3389/fcvm.2022.989243

**Published:** 2022-10-13

**Authors:** Ying Yuan, Hong Qiu, Xiaoying Hu, Jun Zhang, Yuan Wu, Shubin Qiao, Yuejin Yang, Runlin Gao

**Affiliations:** ^1^Department of Cardiology, The First Affiliated Hospital of Sun Yat-sen University, Guangzhou, China; ^2^Fuwai Hospital, National Center for Cardiovascular Diseases, Chinese Academy of Medical Sciences & Peking Union Medical College, Beijing, China

**Keywords:** acute kidney injury, contrast, risk score model, percutaneous coronary intervention, emergency procedure

## Abstract

**Background:**

The previously built score models of contrast-induced acute kidney injury (CI-AKI) were principally founded on selective percutaneous coronary intervention (PCI) cases. Our study was to form a risk score model of CI-AKI and make a temporal validation in a population who underwent emergency PCIs.

**Methods:**

We included patients who underwent emergency PCIs from 2013 to 2018 and divided them into the derivation and validation cohorts. Logistic regression analysis was harnessed to create the risk model. In this research, we defined CI-AKI as an increase in serum creatinine (SCr) ≥0.5 mg/dL (44.2 μmol/L) above baseline within seven days following exposure to contrast medium.

**Results:**

A total of 3564 patients who underwent emergency PCIs were enrolled and divided into the derivation (2376 cases) and validation cohorts (1188 cases), with CI-AKI incidence of 6.61 and 5.39%, respectively. By logistic analysis, the CI-AKI risk score model was constituted by 8 variables: female (1 point), history of transient ischemic attack (TIA)/stroke (1 point), left ventricular ejection fraction (LVEF) classification (1 point per class), big endothelin-1 (ET-1) classification (1 point per class), estimated glomerular filtration rate (eGFR) classification (1 point per class), intra-aortic balloon pump (IABP) application (1 point), left anterior descending (LAD) stented (1 point), and administration of diuretic (2 points). The patients could be further divided into three groups: low-risk, moderate-risk, and high-risk groups, in accordance with the risk scores of 3–6, 7–10, and ≥11 points, and to the CI-AKI rates of 1.4, 11.9, and 42.6%. The CI-AKI risk score model performed well in discrimination (*C* statistic = 0.787, 95% CI: 0.731–0.844) and calibration ability, and showed a superior clinical utility.

**Conclusion:**

We developed a simple CI-AKI risk score model which performs well as a tool for CI-AKI prediction in patients who underwent emergency PCIs.

## Introduction

It has witnessed the wide application of percutaneous coronary intervention (PCI) in coronary artery disease (CAD) patients. It is reported that contrast-induced acute kidney injury (CI-AKI), as one of the serious complications of PCI and the third leading cause of AKI in hospitalized patients, is linked to increased morbidity and mortality (1). During the past decades, the CI-AKI incidence has been generally reported to stay at the range of 2–30% depending on different study populations and various CI-AKI definitions (2, 3). The pathogenesis of CI-AKI has not been wholly elucidated (4, 5), and there are no definitively effectual strategies for prophylaxis or treatment of CI-AKI clinically (6–11). The identification has been made of a strong correlation between CI-AKI and adverse clinical outcomes, particularly in those with acute coronary syndrome (ACS) (12). Therefore, importance should be attached to emergency PCI cases in terms of the high risk of CI-AKI and restricted prophylactic strategies (13). Several clinical prediction models for CI-AKI have been created to anticipate the possibility of CI-AKI after PCI procedures, however, most of which were based on populations who underwent selective PCIs (14–17). The risk factors in the models might not be applicable to emergency cases (8), and the risk factor profile and its cumulative effect for CI-AKI in emergency PCI patients have not been well-studied in a large cohort. The present research aimed to establish a risk score model of CI-AKI and make a temporal validation in an emergency PCI population.

## Methods

### Study population

This observational and retrospective clinical research initially enrolled the patients who suffered emergency PCIs at Fuwai Hospital, Beijing, China from 2013 to 2018. The enrollment criterion was that patients suffered an emergency PCI procedure for suspicious ACS; the exclusion criteria were that patients (1) underwent coronary angiography only, (2) had been applied to by contrast medium <1 week prior to the procedure, (3) had a door-to-balloon time longer than 72 h, (4) had missing or mistaken information/data, (5) underwent procedures on peripheral arteries, (6) had end stage renal disease (ESRD), dialysis, shock, malignant carcinoma, severe liver disease, acute or chronic infection, or autoimmune disease, or (7) declined to participate in the research.

The study protocol conforms to the ethical guidelines of the 1975 *Declaration of Helsinki*. The study was approved by Ethics Committee of Fuwai Hospital, National Center for Cardiovascular Diseases, Chinese Academy of Medical Sciences & Peking Union Medical College. The requirement of such a retrospective research in nature for the informed consent was waived.

### Study endpoint

The study endpoint was the occurrence of CI-AKI during hospitalization. In the study, we defined CI-AKI as an increase in SCr ≥0.5 mg/dL (44.2 μmol/L) above baseline within seven days following exposure to contrast medium (18).

### Study protocol

The patients who conformed to the inclusion and exclusion criteria were finally enrolled and divided into two groups in a 2:1 manner chronologically (19): the development cohort to form the risk score model, and the validation cohort to test it. The clinical data of the population were obtained from the medical records. PCI strategy and periprocedural medications were rested upon the current guidelines. Hydration began as soon as possible after admission, and continued until 18 to 24 h after the procedure with 1 ml·kg^−1^·h^−1^ of normal saline (20, 21). Non-ionic, low or iso-osmolar contrast medium was applied during the procedure. SCr was routinely tested on admission and daily within seven days post the procedure. The left ventricular ejection fraction (LVEF) was tested and the big endothelin-1 (big ET-1) was assayed post the procedure. The patients were discharged more than seven days after the procedure. The calculation of estimated glomerular filtration rate (eGFR) was conducted by CKD-EPI (Chronic Kidney Disease Epidemiology Collaboration) equation (22).

### Statistical analysis

We summarized the baseline characteristics as mean ± standard deviation (SD) or median with interquartile range (IQR) for the continuous variables, and count and percentage for the categorical variables. In the derivation cohort, univariable and multivariable logistic regression analyses were employed to pinpoint the independent predictors of CI-AKI and created the CI-AKI risk model. A bootstrapped study was performed by selecting a total of 1000 bootstrapping samples from the derivation set to evaluate stability and potential model overfit (23). The CI-AKI risk score was created by assigning a weighted integer coefficient value to each independent risk factor and calculating the sum of the coefficients. According to the population distribution and risk score assignment, a three-leveled risk stratification was developed. The trend of CI-AKI rate by risk score and risk stratification was presented in a line graph fitted using the generalized additive model or Loess regression model. In the validation cohort, the CI-AKI risk score model was tested in the discrimination and calibration powers, with the former measured using *C* statistic and the latter using calibration curve (23). *C* statistic addresses the degree to which a model predicts a higher possibility of having an event among patients who will have an event in contrast to those who will not. Calibration plot graphicly represents the link between the observed outcome frequencies and the predicted probabilities, with a bootstrapped analysis of 1000 resamplings of the group (24). During the verification of the risk score model, the calculation of the total points of each patient was made, and the logistic regression analysis was carried out with the total points as a risk factor. Consequently, the *C* statistic and calibration curve were derived based on the logistic regression analysis (25). A decision curve analysis was performed to assess the clinical implications of the model (26), and the performance of our model was compared to Mehran's contrast-induced nephropathy (CIN) risk score model which was based on a population suffering PCIs with 35.7% of patients with ACS (14). As this analysis shows, the clinical benefit was assessed by the “net benefit”: a value of zero suggests no benefit, while a higher value signifies a greater benefit (26).

A two-sided *P*-value <0.05 was considered to indicate a statistical significance. All statistical analyses were performed using the IBM SPSS Statistics Version 22 (IBM, Armonk, NY, USA) and RStudio version 1.2.1335 (Boston, MA, USA).

## Results

### Study population

The study initially included 5807 patients in total, among which, 971 only underwent coronary angiography, 632 underwent selective procedures, 249 had been applied to with contrast medium <7 days prior to the procedure, 197 had a door-to-balloon time longer than 72 h, 100 had a serious cardiogenic shock or died, 66 had mistaken or missing information/data, 19 underwent procedures on peripheral arteries, 7 had malignant cancers, and 2 refused to participate in the research. Ultimately, a total of 3564 cases were enrolled and divided into the derivation and validation cohorts in a 2:1 manner chronologically (19), with the CI-AKI incidence of 6.61% (157 of 2376 patients) and 5.39% (64 of 1188 patients), respectively, as depicted in Supplementary Figure 1.

### Baseline characteristics

The baseline clinical features of patients in the derivation cohort are displayed in Table 1, from which the age was 59 ± 12 years, while the age of CI-AKI patients was 65 ± 12 years. Female patients accounted for 21% in the cohort and 38% in the CI-AKI group. The mean of LVEF was 54%, and eGFR was 86 ml·min^−1^·1.73 m^−2^ in the cohort, while in the CI-AKI patients, the values were 47% and 74 ml·min^−1^·1.73m^−2^, respectively. The median level of the big ET-1 was 0.32 pmol/L in the cohort and 0.48 pmol/L in the CI-AKI cases. The variables LVEF, big ET-1, and eGFR were classified clinically and statistically, and the clinical traits of the classified variables are also given in Table 1. The baseline characteristics of patients in the validation cohort are displayed in Supplementary Table 1.

**Table 1:** Clinical characteristics in the derivation cohort.

**Variable**	**The cohort** **(n = 2,376)**	**CI-AKI** **(n = 157)**	**Non-CI-AKI** **(n = 2,219)**
Age (years)	59.2 ± 11.9	64.9 ± 12.4	58.8 ± 11.8
Female	499 (21.0)	60 (38.2)	439 (19.8)
Height (cm)	168 ± 7	166 ± 8	169 ± 7
Body weight (kg)	73.8 ± 12.6	70.9 ± 13.3	74.0 ± 12.5
BSA (m^2^)	1.82 ± 0.18	1.77 ± 0.19	1.83 ± 0.18
Smoking	1579 (66.5)	92 (58.6)	1487 (67.0)
Hypertension	1446 (60.9)	115 (73.2)	1331 (60.0)
Hyperlipidemia	1869 (78.7)	126 (80.3)	1743 (78.5)
DM	680 (28.6)	51 (32.5)	629 (28.3)
History of MI	339 (14.3)	34 (21.7)	305 (13.7)
History of TIA/stroke	352 (14.8)	43 (27.4)	309 (13.9)
SBP (mmHg)	125 ± 18	127 ± 21	125 ± 17
DBP (mmHg)	75 ± 13	75 ± 14	75 ± 13
LVEF (%)	54 ± 7	47 ± 8	55 ± 7
LVEF classification			
1. LVEF ≥ 50	1826 (76.9)	60 (38.2)	1766 (79.6)
2. 40 ≤ LVEF < 50	449 (18.9)	70 (44.6)	379 (17.1)
3. 30 ≤ LVEF < 40	99 (4.2)	27 (17.2)	72 (3.2)
4. LVEF < 30	2 (0.1)	0 (0)	2 (0.1)
WBC (×10^9^/L)	10.7 ± 3.3	11.5 ± 4.0	10.6 ± 3.2
Hb (×10^12^/L)	145 ± 17	140 ± 19	146 ± 17
Platelet (×10^9^/L)	225 ± 64	224 ± 58	225 ± 64
Fasting glucose (mmol/L)	6.8 [5.5, 8.8]	7.2 [6.0, 9.5]	6.7 [5.5, 8.7]
LDL-c (mmol/L)	2.85 ± 0.92	2.78 ± 0.97	2.85 ± 0.91
hs-CRP (mg/L)	6.5 [2.6, 11.8]	10.6 [5.2, 12.7]	6.2 [2.6, 11.7]
Big ET-1 (pmol/L)	0.32 [0.22, 0.44]	0.48 [0.31, 0.74]	0.31 [0.22, 0.42]
Big ET-1 classification			
1. Big ET-1 < 0.5	1913 (80.5)	81 (51.6)	1832 (82.6)
2. 0.5 ≤ Big ET-1 < 1.0	365 (15.4)	53 (33.8)	312 (14.1)
3. 1.0 ≤ Big ET-1 < 1.5	62 (2.6)	15 (9.6)	47 (2.1)
4. Big ET-1 ≥ 1.5	36 (1.5)	8 (5.1)	28 (1.3)
SCr (μmol/L)	83 ± 23	94 ± 38	82 ± 21
eGFR (ml·min^−1^·1.73 m^−2^)	86 ± 19	74 ± 24	87 ± 19
eGFR classification			
1. eGFR ≥ 90	1142 (48.1)	47 (29.9)	1095 (49.3)
2. 60 ≤ eGFR < 90	976 (41.1)	66 (42.0)	910 (41.0)
3. 30 ≤ eGFR < 60	233 (9.8)	37 (23.6)	196 (8.8)
4. 15 ≤ eGFR < 30	25 (1.1)	7 (4.5)	18 (0.8)
5. eGFR < 15	0 (0)	0 (0)	0 (0)
Onset-to-balloon time (h)	7 [5, 12]	8 [5, 13]	7 [5, 12]
IABP implantation	250 (10.5)	50 (31.8)	200 (9.0)
LAD impaired	2,030 (85.4)	138 (87.9)	1,892 (85.3)
LAD stented	1,570 (66.1)	127 (80.9)	1,443 (65.0)
Contrast volume (mL)	170 [100, 200]	170 [100, 200]	170 [110, 200]
β-blocker	2,083 (87.7)	143 (91.1)	1,940 (87.4)
ACEI/ARB	1,862 (78.4)	113 (72.0)	1,749 (78.8)
Diuretic	909 (38.3)	129 (82.2)	780 (35.2)
Statin	2,358 (99.2)	154 (98.1)	2,204 (99.3)

CI-AKI, contrast-induced acute kidney injury; BSA, body surface area; DM, diabetes mellitus; MI, myocardial infarction; TIA, transient ischemia attack; SBP, systolic blood pressure; DBP, diastolic blood pressure; LVEF, left ventricular ejection fraction; WBC, white blood cell; Hb, hemoglobin; LDL-c, low-density lipoprotein cholesterol; hs-CRP, high-sensitive C-reactive protein; Big ET-1, big endothelin-1; SCr, serum creatinine; eGFR, estimated glomerular filtration rate; IABP, intra-aortic balloon pump; LAD, left anterior descending; ACEI, angiotensin-converting enzyme inhibitor; ARB, angiotensin II receptor blocker.

### Logistic regression analysis

By univariable logistic analysis, 21 variables showed statistical significance for CI-AKI, which were age, female, height, body weight, body surface area (BSA), smoking, hypertension, history of myocardial infarction (MI), history of transient ischemic attack (TIA)/stroke, LVEF classification, white blood cell (WBC) count, hemoglobin (Hb), fasting glucose, high-sensitive C-reactive protein (hs-CRP), big ET-1 classification, SCr, eGFR classification, intra-aortic balloon pump (IABP) application, left anterior descending (LAD) stented, and administration of angiotensin-converting enzyme inhibitor (ACEI)/Angiotensin II receptor blocker (ARB) and diuretic, as presented in Supplementary Table 2.

Among the statistically significant risk factors for CI-AKI, 8 variables eventually included in the CI-AKI prediction model after multivariable logistic analysis, including female (odds ratio [OR] 2.012, 95% confidence interval [CI]: 1.378–2.939, *P* < 0.001), history of TIA/stroke (OR 1.738, 95% CI: 1.144–2.640, *P* = 0.01), LVEF classification (OR 1.725, 95% CI: 1.312–2.268, *P* < 0.001), big ET-1 classification (OR 1.680, 95% CI: 1.328–2.125, *P* < 0.001), eGFR classification (OR 1.456, 95% CI: 1.136–1.867, *P* = 0.003), IABP application (OR 1.674, 95% CI: 1.079–2.598, *P* = 0.021), LAD stented (OR 1.934, 95% CI: 1.210–3.090, *P* = 0.006), and administration of diuretic (OR 4.198, 95% CI: 2.623–6.718, *P* < 0.001), as shown in Table 2. Height, body weight, WBC, hs-CRP, and SCr were excluded for multicollinearity in the analysis.

**Table 2:** Multivariable logistic analysis for CI-AKI.

Variable^*^	**β**	**OR**	**95% CI**	***P***
Female	0.699	2.012	1.378–2.939	<0.001
History of TIA/stroke	0.553	1.738	1.144–2.640	0.010
LVEF classification	0.545	1.725	1.312–2.268	<0.001
Big ET-1 classification	0.519	1.680	1.328–2.125	<0.001
eGFR classification	0.376	1.456	1.136–1.867	0.003
IABP implantation	0.515	1.674	1.079–2.598	0.021
LAD stented	0.659	1.934	1.210–3.090	0.006
Diuretic	1.435	4.198	2.623–6.718	<0.001

* Height, body weight, white blood cell, high-sensitive C-reactive protein, and serum creatinine were excluded for multicollinearity.

β, logistic correlation coefficient; OR, odds ratio; CI, confidence interval; CI-AKI, contrast-induced acute kidney injury; TIA, transient ischemia attack; LVEF, left ventricular ejection fraction; Big ET-1, big endothelin-1; eGFR, estimated glomerular filtration rate; IABP, intra-aortic balloon pump; LAD, left anterior descending.

### Model development

**Figure 1:**
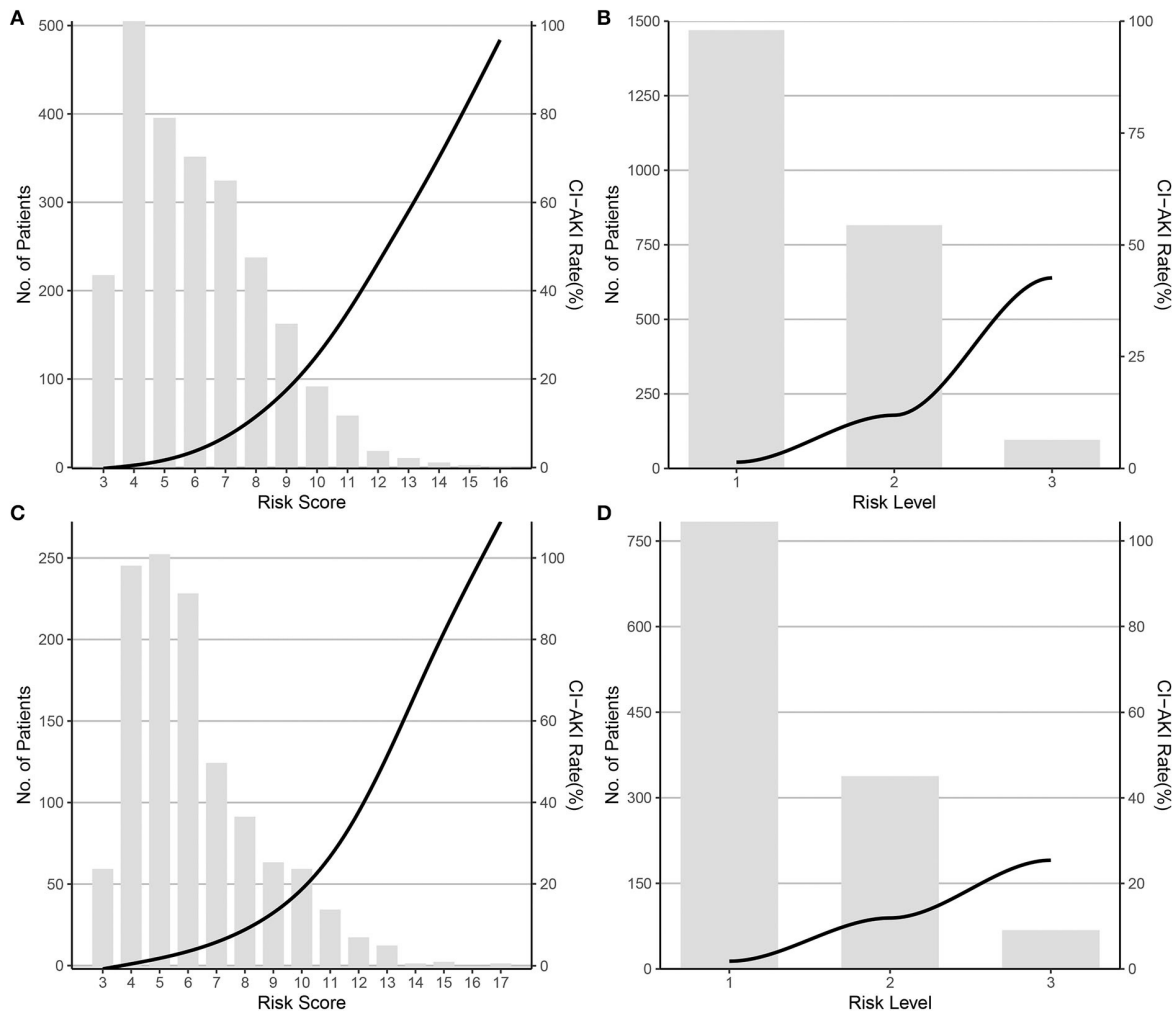
CI-AKI rate by risk score and risk level in the derivation and validation cohort. **(A)** By risk score in the derivation cohort; **(B)** By risk level in the derivation cohort; **(C)** By risk score in the validation cohort; **(D)** By risk level in the validation cohort. CI-AKI, contrast-induced acute kidney injury.

Considering the statistically significant variables as independent risk factors for CI-AKI, a risk scoring system was formed by giving a weighted integer of 1 to each 2 value of OR for each variable/each class of the classified variables. The variables and weighted points were female (1 point), history of TIA/stroke (1 point), LVEF classification (1 point per class), big ET-1 classification (1 point per class), eGFR classification (1 point per class), IABP application (1 point), LAD stented (1 point), and administration of diuretic (2 points). The sum of the weighted coefficients was calculated as the final risk score with a scale of 3–19 points, as shown in Table 3. Founded on the relationship between the frequency distribution and the risk score, the patients could be further divided into three groups: low-risk, moderate-risk, and high-risk groups, in accordance with the risk scores of 3–6, 7–10, and ≥11 points, and to the CI-AKI rates (%) of 1.36 (0.83–2.10), 11.92 (9.87–14.32), and 42.55 (32.41–53.18), as predicted in Supplementary Table 3. The trend of CI-AKI rate by risk score and risk level in the derivation cohort is presented in Figures 1A,B. In the validation cohort, the CI-AKI risk score was calculated according to the formed risk score model and the risk level was assigned according to the risk level categorizing method, and the trend of CI-AKI rate by risk score and risk level in the validation cohort is presented in Figures 1C,D.

**Table 3:** Risk score assignment.

**Variable**	**Score**
Female	1
History of TIA/stroke	1
LVEF classification (%)	
1. LVEF ≥ 50	1
2. 40 ≤ LVEF < 50	2
3. 30 ≤ LVEF < 40	3
4. LVEF < 30	4
Big ET-1 classification (pmol/L)	
1. Big ET-1 < 0.5	1
2. 0.5 ≤ Big ET-1 < 1.0	2
3. 1.0 ≤ Big ET-1 < 1.5	3
4. Big ET-1 ≥ 1.5	4
eGFR classification (ml·min^−1^·1.73m^−2^)	
1. eGFR ≥ 90	1
2. 60 ≤ eGFR < 90	2
3. 30 ≤ eGFR < 60	3
4. 15 ≤ eGFR < 30	4
5. eGFR < 15	5
IABP implantation	1
LAD stented	1
Diuretic	2
Range	3–19

TIA, transient ischemia attack; LVEF, left ventricular ejection fraction; Big ET-1, big endothelin-1; eGFR, estimated glomerular filtration rate; IABP, intra-aortic balloon pump; LAD, left anterior descending.

### Model performance

**Figure 2:**
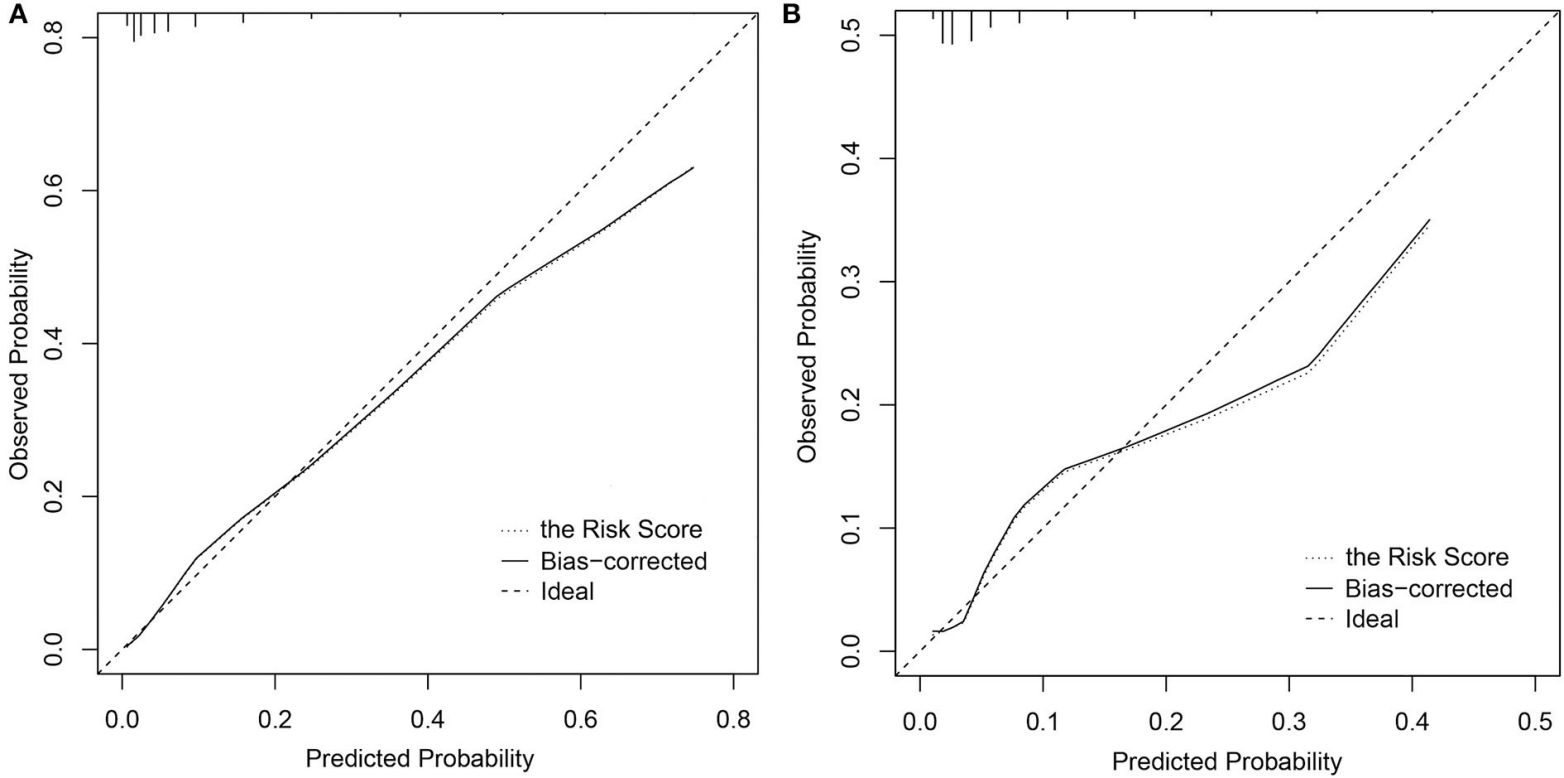
Calibration curves of the CI-AKI risk score model. **(A)** In the derivation cohort; **(B)** In the validation cohort. CI-AKI, contrast-induced acute kidney injury.

The CI-AKI risk score model performed a good discrimination ability in the derivation and validation cohorts with *C* statistic = 0.837, 95% CI: 0.806–0.869 and *C* statistic = 0.787, 95% CI: 0.731–0.844, respectively. In calibration performance, the 1000-sample bootstrapped calibration plot is presented in Figure 2, from which a good rapport was exhibited on the probability of CI-AKI between the prediction and observation in the derivation and validation cohorts.

### Clinical utility

**Figure 3:**
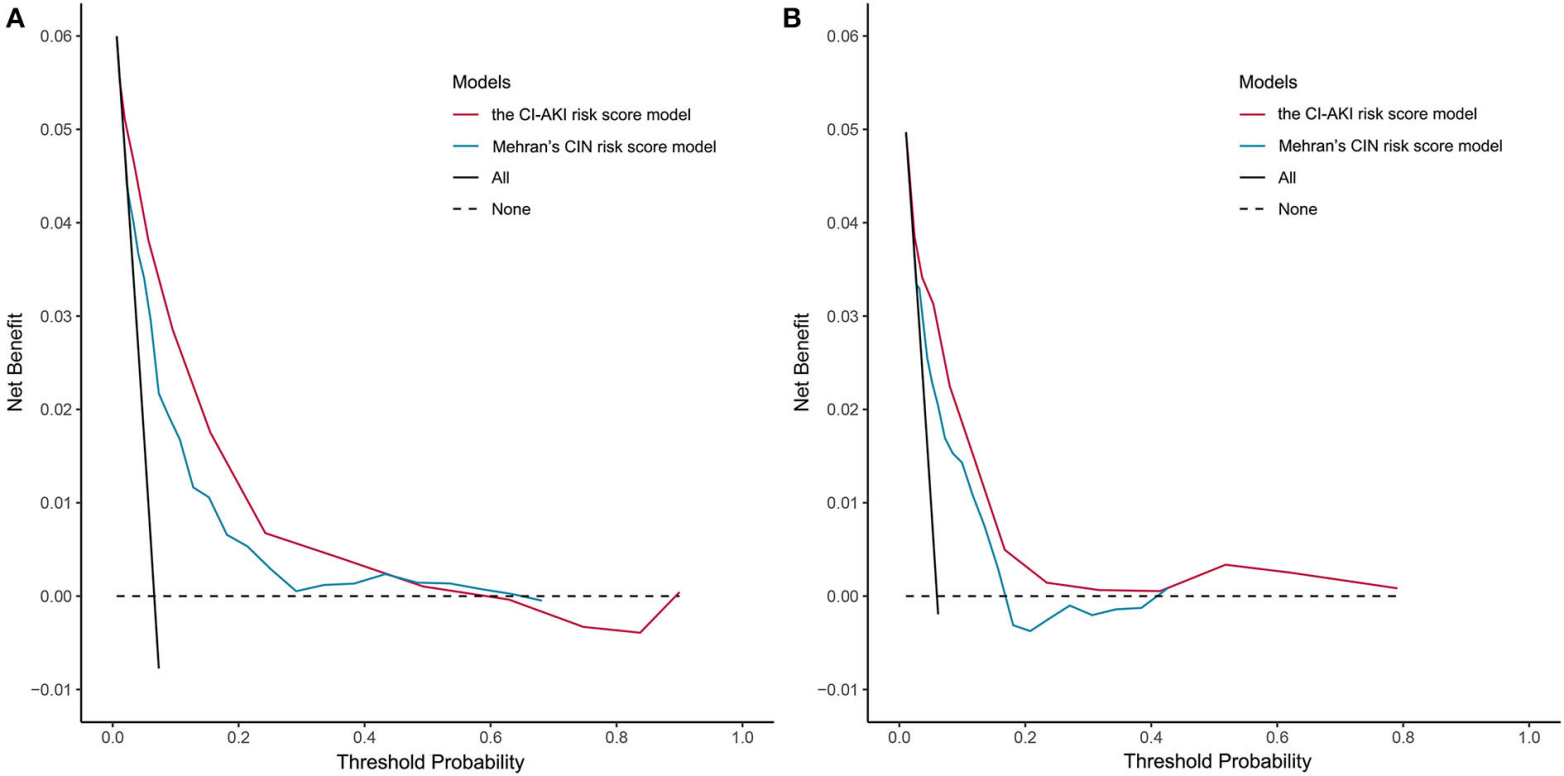
Decision curve analysis of the CI-AKI risk score model. **(A)** In the derivation cohort; **(B)** In the validation cohort. CI-AKI, contrast-induced acute kidney injury.

The results of the decision curve analysis used to examine the clinical utility are illustrated in Figure 3, from which, the net benefit of our risk score model was over zero in the threshold probability (*Pt*) of <60%, and the clinical benefit of our risk score model was superior to Mehran's CIN risk score model in the *Pt* of <40%, both in the derivation and validation cohorts.

## Discussion

During the last decades, CI-AKI has been reported as one of the most serious complications in patients suffering emergency PCIs, especially in those with ACS (12). The emphasis should be placed on emergency cases with a high possibility of CI-AKI and restricted preventive strategies (13). Most of the published CI-AKI risk score models were based on populations who underwent selective PCIs (14–17), and there is no study to form a CI-AKI risk score model in a population who underwent emergency PCIs. Our project created a risk score model of CI-AKI and made a temporal validation in such a population. The model consists of eight available variables which are female (1 point), history of TIA/stroke (1 point), LVEF classification (1 point per class), big ET-1 classification (1 point per class), eGFR classification (1 point per class), IABP application (1 point), LAD stented (1 point), and administration of diuretic (2 points). The patients who underwent emergency PCIs could be further categorized three folds: low-risk, moderate-risk, and high-risk groups, corresponding to the risk scores of 3–6, 7–10, and ≥11 points, and to the CI-AKI rates of 1.4, 11.9, and 42.6%.

In the study, we defined the CI-AKI as an absolute rise of SCr ≥44.2 μmol/L (0.5 mg/dL) over baseline within 7 days following the exposure to contrast agent (18). The debate around optimal definition of CI-AKI has last for decades (2, 27). Harjai et al. discovered that both a relative increase in SCr ≥25% and an absolute increase of SCr ≥44.2 μmol/L were independently related to poorer clinical outcomes, with the absolute cutoff value more evident (28). As Crimi et al. revealed, under the CI-AKI definition of an SCr increase ≥25%, a low relevance between risk factors and CI-AKI occurrence was shown, especially in emergency cases (8). Based on the results of our previous studies, under the CI-AKI definition of a relative SCr increase ≥25%, a confusing conclusion was made that a lower baseline SCr was more associated with CI-AKI development, probably as a result of the high sensitivity of the definition (29, 30). Mehran and colleagues revealed that the definition of small increments in SCr is neither limited to injury by the application of contrast material nor specifically suggestive of inherent kidney damage (31). Therefore, a relatively large cutoff of SCr increase ≥44.2 μmol/L in the CI-AKI definition was used in the study. On the other side, concerning the cutoff time, our study set at 7 days post-procedure, different from the time point at 72 h as some investigations made (32). Although CI-AKI typically manifests within 72 h after the PCI procedures, it might not be an appropriate time-point because it has been announced that CI-AKI often culminates within 3 to 5 days and then declines within 10 to 21 days (4, 33). Hence, CI-AKI for emergency PCI population might be appropriately defined by an absolute increase in SCr ≥44.2 μmol/L within 7 days post the procedure, which was ultimately used in the study.

The risk factor profile of the CI-AKI risk model covers aspects of demographics, patient history, baseline cardiac and renal function, vasculature condition, procedural characteristics, and medicine administration. In the model, one point is assigned to each variable/each class of classified variable with the exception of diuretic administration of two points. The present study showed that female gender was independently at higher risk of CI-AKI consistent with the prior studies (34, 35), which could be attributed to the effect of ovarian hormones on the renin-angiotensin system and the renal blood flow (36). Different from previous studies, big ET-1 and administration of diuretic showed significance for CI-AKI in our research. Big ET-1, the proendothelin of ET-1, played a great role in the initial stage of endothelin impairment during the development of CI-AKI (4), and led to the possible emergence of hemodynamic instability, especially in those with renal microcirculation change (37). Given the administration of diuretic, experimental data indicate that renal circulation can be changed after diuretic contact resulting in AKI development because of renal hypoxia and inflammatory reactions (38). Therefore, diuretics should not be recommended for emergency PCI cases due to the high risk of CI-AKI (38).

The volume of contrast medium did not display any significance for CI-AKI in the study. This result, similar to those of previous researches (39, 40), leads us to conclude that the intravascular application of iodinated contrast agents might not be linked to an increased risk of AKI. Caspi et al. also reported that the risk for AKI was applicable to ST-segment elevation myocardial infarction (STEMI) patients with and without contrast material exposure, arguing that the contact with contrast medium might not be the culprit of AKI (41). Over time, lower rates of AKI after the application of contrast materials occurred as a result of an evolution in the design of contrast agents, improved recognition of risk factors, and implementation of preventive care (2, 31). Since some other risk factors, apart from contrast agent, can precipitate AKI after exposure to contrast medium, it is suggested that CI-AKI should be changed to another favored term “contrast-associated acute kidney injury (CA-AKI)” in Mehran's review (31).

In our study, a CI-AKI risk score model was developed and a temporal validation was made in a population with emergency PCIs. The *C* statistic of 0.787 with 95% CI 0.731–0.844, signifies a concordance of 78.7% between the prediction by the CI-AKI score system and the reality of CI-AKI occurrence, showing a high discriminative power. The calibration curves manifest a good agreement by exhibiting the probability of CI-AKI between the prediction and observation, predicting that it performs well in calibration ability. The clinical utility was also tested with the net benefit of our risk score model over zero in the *Pt* of <60%, and the clinical advantage of the model was superior to that of Mehran's CIN risk score model in the *Pt* of <40%.

Admittedly, several limitations transpire in our research. First, the study was based on patients from a single center and the data were collected retrospectively. Second, the application of periprocedural hydration would exert an influence on the SCr values at the baseline and during the follow-up. Third, we did not enroll severe patients, such as those with ESRD, cardiogenic shock and dialysis, because these diseases would have confounding effect on the occurrence of CI-AKI. Fourth, the numbers of patients in the high-risk groups of the derivation and validation cohorts were sparse. Moreover, the LVEF and the big ET-1 was collected post the procedure, and some variables in the model are not available before the procedure, such as IABP application, LAD stented and administration of diuretic. However, CI-AKI would be likely to occur during a long period post the procedure (33), and it is still of necessity and significance to evaluate the CI-AKI risk even though the procedure has been finished (31). In the study, a CI-AKI risk score model was developed and validated in an emergency PCI population. Further researches should be carried out to explore the power of the CI-AKI risk score system in different population and for different clinical outcomes.

## Conclusion

In our study, a CI-AKI risk score model was developed and validated in a population who suffered emergency PCIs. The suggested score is viable and utilizable, allowing for easy risk assessment practically.

## Data availability statement

The raw data supporting the conclusions of this article will be made available by the authors, without undue reservation.

## Ethics statement

The studies involving human participants were reviewed and approved by Ethics Committee of Fuwai Hospital, National Center for Cardiovascular Diseases, Chinese Academy of Medical Sciences & Peking Union Medical College. Written informed consent for participation was not required for this study in accordance with the national legislation and the institutional requirements.

## Author contributions

YY and HQ designed the study. YY and XH engaged in data collection and verified the accuracy and completeness of the data. YY, HQ, XH, JZ, YW, SQ, YY, and RG interpreted the data. YY analyzed the data and wrote the first draft of the manuscript. HQ, SQ, YY, and RG critically reviewed and revised the manuscript and other authors provided very valuable comments for manuscript revision. All authors contributed to the article and approved the submitted version.

## Conflict of interest

The authors declare that the research was conducted in the absence of any commercial or financial relationships that could be construed as a potential conflict of interest.

## Publisher's note

All claims expressed in this article are solely those of the authors and do not necessarily represent those of their affiliated organizations, or those of the publisher, the editors and the reviewers. Any product that may be evaluated in this article, or claim that may be made by its manufacturer, is not guaranteed or endorsed by the publisher.
