# Supplementary material for: A risk score model of contrast-induced acute kidney injury in patients with emergency percutaneous coronary interventions
Source: Front Cardiovasc Med. 2022 Oct 13;9:989243. doi: 10.3389/fcvm.2022.989243 (PMC9606750; doi:10.3389/fcvm.2022.989243)
Supplement: Supplementary file 1 [file Data_Sheet_1.pdf]

Supplementary Material

Supplementary Figure 1. Patient flowchart.
Supplementary Table 1. Clinical characteristics in the validation cohort.
Supplementary Table 2. Univariable logistic analysis for CI-AKI.
Supplementary Table 3. Risk stratification and CI-AKI rate.

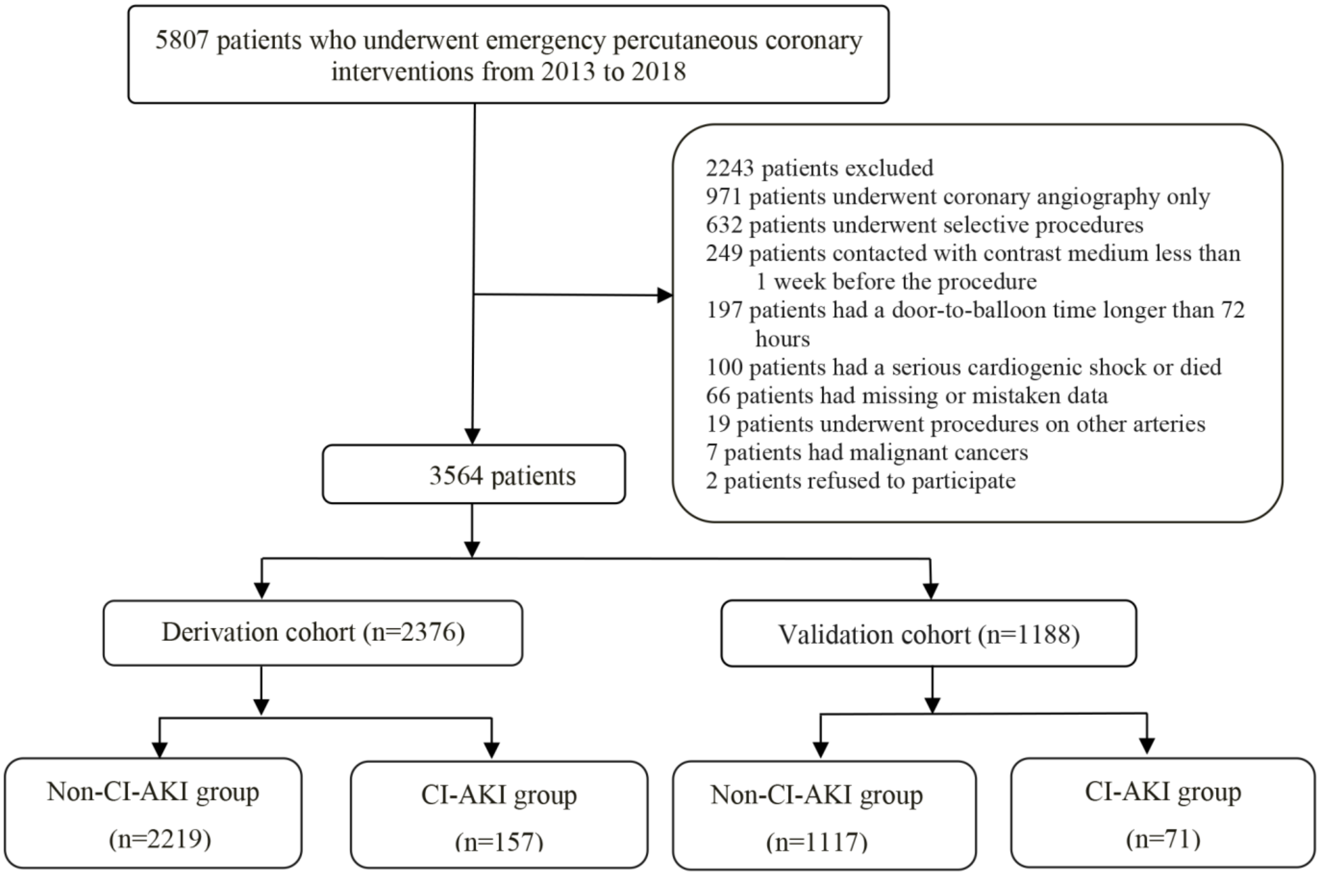

Supplementary Figure 1. Patient flowchart. CI-AKI, contrast-induced acute kidney injury.

Supplementary Table 1. Clinical characteristics in the validation cohort.

Variable	The cohort (n = 1188)	CI-AKI (n = 71)	Non-CI-AKI (n = 1117)
Age (years)	60.1 ± 11.8	62.0 ± 12.3	59.9 ± 11.7
Female	249 (21.0)	17 (23.9)	232 (20.8)
Height (cm)	169 ± 7	167 ± 9	169 ± 7
Body weight (kg)	74.2 ± 12.6	70.9 ± 12.9	74.4 ± 12.5
BSA (m^2)	1.83 ± 0.18	1.78 ± 0.19	1.83 ± 0.18
Smoking	752 (63.3)	38 (53.5)	714 (63.9)
Hypertension	755 (63.6)	56 (78.9)	699 (62.6)
hyperlipidemia	961 (80.9)	60 (84.5)	901 (80.7)
DM	380 (32.0)	26 (36.6)	354 (31.7)
History of MI	205 (17.3)	17 (23.9)	188 (16.8)
History of TIA/stroke	192 (16.2)	14 (19.7)	178 (15.9)
SBP (mmHg)	126 ± 18	127 ± 22	126 ± 18
DBP (mmHg)	78 ± 12	81 ± 14	78 ± 12
LVEF (%)	54 ± 7	48 ± 10	55 ± 7
LVEF classification			
1 LVEF≥50	916 (77.1)	35 (49.3)	881 (78.9)
2 40≤LVEF<50	220 (18.5)	22 (31.0)	198 (17.7)
3 30≤LVEF<40	48 (4.0)	12 (16.9)	36 (3.2)
4 LVEF<30	4 (0.3)	2 (2.8)	2 (0.2)
WBC ($\times 10^9$/L)	10.0 ± 3.2	10.1 ± 3.1	10.0 ± 3.2
Hb ($\times 10^{12}$/L)	146 ± 17	143 ± 23	146 ± 17
Platelet ($\times 10^9$/L)	232 ± 70	238 ± 84	232 ± 69
Fasting glucose (mmol/L)	6.4 [5.3, 8.4]	6.9 [6.2, 9.3]	6.4 [5.3, 8.3]
LDL-c (mmol/L)	2.70 ± 0.90	2.59 ± 0.86	2.71 ± 0.90
hs-CRP (mg/L)	5.69 [2.21, 10.47]	7.98 [1.82, 12.00]	5.63 [2.22, 10.34]
Big ET-1 (pmol/L)	0.40 [0.26, 0.60]	0.65 [0.32, 0.96]	0.39 [0.26, 0.58]
Big ET-1 classification			
1 Big ET-1<0.5	770 (64.8)	25 (35.2)	745 (66.7)
2 0.5≤Big ET-1<1.0	327 (27.5)	30 (42.3)	297 (26.6)
3 1.0≤Big ET-1<1.5	53 (4.5)	6 (8.5)	47 (4.2)
4 Big ET-1≥1.5	38 (3.2)	10 (14.1)	28 (2.5)
SCr (μmol/L)	82 ± 25	94 ± 33	81 ± 24
eGFR (ml/min·1.73m^2)	86 ± 19	76 ± 23	86 ± 19
eGFR classification			
1 eGFR≥90	573 (48.2)	21 (29.6)	552 (49.4)
2 60≤eGFR<90	492 (41.4)	31 (43.7)	461 (41.3)
3 30≤eGFR<60	112 (9.4)	16 (22.5)	96 (8.6)
4 15≤eGFR<30	10 (0.8)	3 (4.2)	7 (0.6)
5 eGFR<15	1 (0.1)	0 (0)	1 (0.1)
Onset-to-balloon time (h)	8 [5, 15]	8 [5, 15]	8 [5, 15]
IABP implantation	96 (8.1)	20 (28.2)	76 (6.8)
LAD impaired	1012 (85.2)	60 (84.5)	952 (85.2)
LAD stented	1007 (84.8)	68 (95.8)	939 (84.1)
Contrast volume (mL)	180 [160, 190]	180 [160, 200]	180 [160, 190]
β-blocker	1032 (86.9)	66 (93.0)	966 (86.5)

ACEI/ARB	904 (76.1)	51 (71.8)	853 (76.4)
Diuretic	300 (25.3)	48 (67.6)	252 (22.6)
Statin	1182 (99.5)	68 (95.8)	1114 (99.7)

CI-AKI, contrast-induced acute kidney injury; BSA, body surface area; DM, diabetes mellitus; MI, myocardial infarction; TIA, transient ischemia attack; SBP, systolic blood pressure; DBP, diastolic blood pressure; LVEF, left ventricular ejection fraction; WBC, white blood cell; Hb, hemoglobin; LDL-c, low-density lipoprotein cholesterol; hs-CRP, high-sensitive C-reactive protein; Big ET-1, big endothelin-1; SCr, serum creatinine; eGFR, estimated glomerular filtration rate; IABP, intra-aortic balloon pump; LAD, left anterior descending; ACEI, angiotensin-converting enzyme inhibitor; ARB, angiotensin II receptor blocker.

Supplementary Table 2. Univariable logistic analysis for CI-AKI.

Variable	*β*	OR	95% CI	*P*
Age	0.044	1.045	1.030-1.060	<0.001
Female	0.920	2.508	1.788-3.518	<0.001
Height	-0.050	0.951	0.932-0.972	<0.001
Body weight	-0.020	0.980	0.967-0.993	0.003
BSA	-1.720	0.179	0.074-0.431	<0.001
Smoking	-0.361	0.697	0.501-0.969	0.032
Hypertension	0.603	1.827	1.270-2.627	0.001
History of MI	0.551	1.735	1.165-2.584	0.007
History of TIA/stroke	0.847	2.332	1.609-3.378	<0.001
LVEF classification	1.272	3.567	2.849-4.466	<0.001
WBC	0.071	1.073	1.025-1.123	0.002
Hb	-0.018	0.983	0.974-0.991	<0.001
Fasting glucose	0.083	1.087	1.041-1.135	<0.001
hs-CRP	0.095	1.100	1.065-1.136	<0.001
Big ET-1 classification	0.848	2.335	1.931-2.823	<0.001
SCr	0.015	1.015	1.010-1.021	<0.001
eGFR classification	0.735	2.086	1.696-2.566	<0.001
IABP implantation	1.551	4.717	3.272-6.801	<0.001
LAD stented	0.823	2.277	1.515-3.421	<0.001
ACEI/ARB	-0.371	0.690	0.480-0.992	0.045
Diuretic	2.140	8.500	5.597-12.908	<0.001

β, logistic correlation coefficient; OR, odds ratio; CI, confidence interval; CI-AKI, contrast-induced acute kidney injury; BSA, body surface area; MI, myocardial infarction; TIA, transient ischemia attack; LVEF, left ventricular ejection fraction; WBC, white blood cell; Hb, hemoglobin; hs-CRP, high-sensitive C-reactive protein; Big ET-1, big endothelin-1; SCr, serum creatinine; eGFR, estimated glomerular filtration rate; IABP, intra-aortic balloon pump; LAD, left anterior descending; ACEI, angiotensin-converting enzyme inhibitor; ARB, angiotensin II receptor blocker.

Supplementary Table 3. Risk stratification and CI-AKI rate.

Risk level	Score*	CI-AKI rate (%)	95% CI
Low risk	3-6	1.36	0.83-2.10
Moderate risk	7-10	11.92	9.87-14.32
High risk	≥11	42.55	32.41-53.18

*The score ranges 3-19 points.

CI-AKI, contrast-induced acute kidney injury; CI, confidence interval.
